# Modified PEHPS Medium as an Alternative for the *In Vitro* Culture of *Giardia lamblia*

**DOI:** 10.1155/2014/714173

**Published:** 2014-05-21

**Authors:** Javier Vargas-Villarreal, Benito D. Mata-Cárdenas, Magda E. Hernández-García, Jesús N. Garza-González, Laura H. De La Garza-Salinas, Francisco González-Salazar

**Affiliations:** ^1^Laboratorio de Bioquímica y Fisiología Celular, División de Biología Celular y Molecular, Centro de Investigación Biomédica del Noreste, Instituto Mexicano Del Seguro Social, Administración de Correo No. 4, Apartado Postal 020-E Colonia Independencia, 64720 Monterrey, NL, Mexico; ^2^Facultad de Ciencias Químicas, Universidad Autónoma de Nuevo León, Avenida Universidad s/n, Ciudad Universitaria, 66451 San Nicolás de los Garza, NL, Mexico; ^3^Facultad de Ciencias Biológicas, Universidad Autónoma de Nuevo León, Avenida Pedro de Alba y Manuel L. Barragán s/n, Ciudad Universitaria, 66451 San Nicolás de los Garza, NL, Mexico; ^4^Coordinador Auxiliar Medico, Instituto Mexicano Del Seguro Social, Delegación Nuevo León, Gregorio Torres de Quevedo No. 1950 Ote, 64000 Monterrey, NL, Mexico

## Abstract

Commercial culture media present interlot variations in biological activity. We have previously designed a homemade and economic culture medium, PEHPS medium, for the axenic cultivation of *Entamoeba histolytica* and *Trichomonas vaginalis*. Trophozoites of amoebae and trichomonads grow well in this medium. Furthermore, the medium is stable for several months when stored frozen or refrigerated. The objective of this work was to modify PEHPS medium to support the *in vitro* growth of *Giardia lamblia*. Inocula of 5 × 10^3^ trophozoites/mL of *G. lamblia* were incubated at 36.5°C in modified PEHPS or TYI-S-33 medium. Then, the growths of the three *Giardia* strains in both media were compared. The logarithmic growth phase lasted 72 h; the mean yield of the strains ranged from 10.06 to 11.43 × 10^5^ *Giardia* trophozoites/mL, and the range of duplication time in the three strains was from 5.67 to 6.06 in modified PEHPS medium. These growth characteristics were not significantly different from those obtained with TYI-S-33 medium. We conclude that modified PEHPS medium might be used for the axenic cultivation of *G. lamblia*.

## 1. Introduction

Several culture media have been developed to facilitate the* in vitro* study of microorganisms. Ideally, the growth and development of the parasites cultivated in these media should be similar to those in their natural habitat [1]. In order to introduce new culture media for the cultivation of protozoa, their applicability for diagnosis and their biochemical and immunological characteristics should be revised [2]. Protozoan flagellate* Giardia lamblia* has been recognized as an important cause of diarrhea and malnutrition in the world [3–6], and its study has been facilitated by the design of several growth media, including the manufactured TYI-S-33 [7]. On the other hand, PEHPS medium was designed in our laboratory more than 25 years ago [8]. This medium was designed by the need to create a homemade, economic medium without the interlot variability present in manufactured culture media, ranging from perfect growth in one lot to hardly any growth in another [1]. The PEHPS was developed from extracts of liver and pancreas obtained from beef and pork and can be produced in any laboratory. With appropriate storage, PEHPS medium lasts up to 3 years without losing its efficiency [9].

This culture medium has been used to grow* Entamoeba histolytica *and* Trichomonas vaginalis* with results equal to those observed with manufactured culture media [10, 11]. In addition, these microorganisms, when grown in PEHPS medium, retain the activity of virulence factors. PEHPS medium also supports the synthesis of cell duringaxenic encystment in* E. histolytica* [12].

The development of culture media is definitely not a diagnostic tool, since there are diagnostic methods that are more efficient and faster than cultivation. However, the development of new means of cultivation for intestinal protozoan allows learning about the biology of parasites, their growth rate, virulence factors, susceptibility to new drugs, and the development of resistant strains among others [1].

Modifications of the PEHPS medium have been realized to optimize culture conditions and eliminate possible inhibitory factors [13].

In this study we provide evidence that* G. lamblia* trophozoites grow and develop in modified PEPHS medium as they do in manufactured TYI-S-33.

## 2. Material and Methods

*G. lamblia* strains IMSS:0989, WB, and IMSS:3 were kindly donated by Dr. Roberto Cedillo Rivera from the Centro Médico Nacional Siglo XXI, México City [14]. The parasites were maintained in axenic conditions at 36.5°C by serial subcultivation. Briefly, 5 × 10^3^ trophozoites/mL were placed in 13 × 100 mm borosilicate screw-capped tubes containing 5.6 mL of TYI-S-33 described by Keister [7]; they were then harvested and reseeded every 72 h.

For assays, the parasites were maintained in axenic conditions during 30 days at 36.5°C by serial subcultivation in TYI S 33 [7] or in modified PEHPS medium. Modified PEHPS medium was prepared by supplementing standard PEHPS medium, described by Said-Fernandez et al. [9] (Table 1), with 0.6 g/L bovine bile (Sigma-Aldrich, St Louis, MO, USA) and 10% bovine serum (homemade, see below), and additionally by increasing the cysteine (Sigma-Aldrich, St Louis, MO, USA) and ascorbic acid (Sigma-Aldrich, St Louis, MO, USA) concentrations to 1.26 g/L and 1.00 g/L, respectively (Table 1). The medium was sterilized by filtration over a 0.22 *μ*m filter (Millipore, Millipore Corporation, Billerica, MA, USA) and stored in 5.6 mL aliquots at −20°C. To prepare modified PEHPS medium, bovine bile was added from a 3% w/v bovine bile/phosphate buffered saline (pH 7.3) stock. Bovine serum was homemade. Briefly, fresh blood (about 15 L), from cows for human consumption, was collected in a sterile way at a local slaughterhouse. The serum was separated by blood coagulation and sedimentation. After complement inactivation by heat (56°C for 1 h), the serum was filtered (0.45 or 0.22 *μ*m pores; Millipore, Millipore Corporation, Billerica, MA, USA). The serum was frozen at −70°C until use. For use, 0.5 mL of bovine serum was added in each culture tube containing 5 mL of basal medium. Lots of well-prepared bovine serum took 3 years to be consumed.

The growth of* G. lamblia* in TYI-S-33 or modified PEHPS medium was determined every 24 h for 4 days in triplicate. The number of trophozoites/mL was determined using a haemocytometer, and the doubling time was calculated by linear regression [11]. The yields in each culture were determined at the end of the logarithmic growth phase (72 h of incubation). Yield differences between experimental and reference cultures were assessed by comparing means of assays using the different media by Student's *t*-test. A *P* value <0.05 was considered significative.

## 3. Results

Trophozoites of* G. lamblia* from the 3 strains used in the present study grew immediately after the parasites were transferred from TYI-S-33 to PEHPS, but all strains grew significantly less in modified PEHPS medium as compared to reference cultures (Table 2). The diminishment in yield was 13% for the WB strain, 12.5% for the 0989: IMSS strain, and 7.5% for the IMSS:3 strain.

The doubling times of the three strains grown in modified PEHPS medium were longer than those grown in TYI-S-33 as shown in Table 3.

All strains adapted satisfactorily to allow for growth in modified PEHPS medium.

The addition of bovine bile improved growth substantially. Nevertheless, in our experience, satisfactory yields with TYI-S-33 medium appear to depend on the brand and batch of yeast extract.

## 4. Discussion 

We previously demonstrated that trophozoites of* E. histolytica* and* T. vaginalis* grow well in standard PEHPS medium and that supplementation with bovine bile improved the yield of* E. Histolytica* [10, 13, 15]. The PEHPS medium described by Said-Fernandez et al. [9] does not allow for proper growth of* G. lamblia*. In this paper, we report the modifications to the standard PEHPS medium that allow the adequate growth of* G. lamblia.*

The modified PEHPS medium offers the advantage of being inexpensive and very stable during refrigeration and freezing, so that a large number of experiments can be performed with the same batch of medium for more than 6 months without affecting yields [9].

We provided evidence that* G. lamblia* trophozoites grow well in modified PEHPS medium. The major modifications to the original media were the increase of the concentration of cysteine, ascorbic acid, bile inclusion, and a filtration step over a 0.22 *μ*m membrane. This filtration step not only sterilizes the medium but also may remove certain factors from the medium such as hormones, enzymes, and other proteins from the liver and pancreas extracts [9] that might have been parasite growth inhibitors. The addition of soluble bile factors may favor the synthesis of parasite membranes and virulence factors [16] and therefore may be essential for many biological assays [9].

Increasing the cysteine concentration was justified as an increased cysteine intake by* G. lamblia*  trophozoites that has been reported [17]. Ascorbic acid, present in standard PEHPS at 0.2 g/L [9], was increased to 1 g/L in the modified PEHPS medium in order to ensure the same ascorbic acid concentration that is present inTYI-S-33.

We have maintained an* E. histolytica* strain in continuous culture in PEHPS medium for more than 25 years and* T. vaginalis* for over 15 years. The successful culture of* G. lamblia* in modified PEHPS media allows for the execution of assays in an economical and stable environment, avoiding the risk of variability between assays due to interlot variability that occurs when using commercial culture media [18].

Although* G. lamblia* trophozoites, cultured in modified PEHPS medium, provide lower yields than the ones obtained in TYI-S-33, the yields in modified PEHPS are sufficient to maintain* G. lamblia* strains in a stable and reliable culture for an undefined time. A reliable cultivation of* G. lamblia* in modified PEHPS medium allows us to investigate the biology of this parasite, to study the virulence factors, to develop new better diagnostic method, and to test new drugs for better treatments.

Modified PEHPS medium is an alternative culture medium, which is economic and free of the interlot variability present in manufactured TYI-S-33.

## Figures and Tables

**Table 1:** Comparative ingredients of original PEHPS media [9] with modified PEHPS media.

Compound	Brand	PEHPS [9]	Modified PEHPS
**EHP**	Homemade [9]	250 mL	250 mL
Casein peptone	BD Bioxon	10 g/L	10 g/L
**Ascorbic acid**	**Sigma Aldrich**	**0.2 g/L**	**1 g/L**
**Cysteine**	**Sigma Aldrich**	**1.0 g/L**	**1.26 g/L**
Glucose	Tecnica Quimica	6 g/L	6 g/L
K_2_HPO_4_	J T Baker	1	1
KH_2_PO_4_	Monterrey	0.6	0.6
**Bovine bile**	**Sigma Aldrich**	**0.0 g/L**	**0.6 g/L**
Deionized water		Enough to fill a 1/L	Enough to fill a 1/L

EHP: pancreas-liver extract.

**Table 2:** Yield of *Giardia lamblia* grown in TYI-S-33 or PEHPS medium.

Strain	Medium	Final density	
		Absolute	Relative
WB	TYI-S-33	11.67 ± 0.33	1.00
	PEHPS	10.06 ± 0.32	0.86^†^
0989:IMSS	TYI-S-33	12.52 ± 0.31	1.00
	PEHPS	10.96 ± 0.62	0.88^†^
IMSS:3	TYI-S-33	12.35 ± 0.31	1.00
	PEHPS	11.43 ± 0.43	0.92^†^

Trophozoites ×10^5^ per mL; means ± standard deviations of 8 triplicate experiments.

^†^ *P* > 0.05.

**Table 3:** Doubling times (h) of three strains of *Giardia lamblia* in TYI-S-33 or PEHPS medium.

	TYI-S-33	PEHPS
WB	5.21	6.00
0989:IMSS	4.94	5.67
IMSS:3	4.84	6.06
